# Investigating the associations between uncarboxylated matrix gla protein as a proxy for vitamin K status and cardiovascular disease risk factors in a general adult population

**DOI:** 10.1007/s00394-024-03532-6

**Published:** 2024-11-21

**Authors:** Julie Aaberg Lauridsen, Katja Biering Leth-Møller, Line Tang Møllehave, Line Lund Kårhus, Thomas Meinertz Dantoft, Klaus Fuglsang Kofoed, Allan Linneberg

**Affiliations:** 1 Center for Clinical Research and Prevention, Copenhagen University Hospital, Bispebjerg and Frederiksberg Hospital, Copenhagen, Denmark; 2 Department of Cardiology, Copenhagen University Hospital, Rigshospitalet, Copenhagen, Denmark; 3 Department of Clinical Medicine, Faculty of Health and Medical Sciences, University of Copenhagen, Copenhagen, Denmark

**Keywords:** Vitamin K, Matrix Gla protein, Cardiovascular disease risk factors, Atherosclerosis, Epidemiology

## Abstract

**Purpose:**

Vitamin K is an activator of vitamin K dependent proteins, one of which is the potent inhibitor of vascular calcification, matrix Gla protein (MGP). The purpose of this study is to investigate the association between an inverse proxy of functional vitamin K status, plasma dephospho-uncarboxylated MGP (dp-ucMGP), and cardiovascular disease risk factors (CVDRFs).

**Methods:**

In a cross-sectional population-based health examination study of 4,092 individuals aged 24–77 years, the vitamin K status was assessed using plasma dp-ucMGP. All participants were linked to Danish National Prescription Register to obtain information on the use of vitamin K antagonists. The associations between log2 transformed dp-ucMGP values and CVDRFs were determined using regression models adjusted for sex, age, lifestyle factors, kidney function and waist circumference.

**Results:**

Higher dp-ucMGP levels were associated with increased risk of central obesity (Odds Ratio (OR) 4.76, 95% Confidence Intervals (CI) 3.57–6.34), diabetes (OR 1.96, 95% CI 1.11–3.45), hyperlipidaemia (OR 1.43, 95% CI 1.01–2.03), and impaired kidney function (OR 9.83, 95% CI 5.49–17.59) per doubling in dp-ucMGP. Dp-ucMGP was not independently associated with hypertension or arterial stiffness.

**Conclusion:**

Higher dp-ucMGP levels were associated with central obesity, diabetes, hyperlipidaemia, and impaired kidney function. Prospective studies and intervention studies examining the effects of improving vitamin K status are needed to clarify the potential role of vitamin K in relation to these CVDRFs.

**Supplementary Information:**

The online version contains supplementary material available at 10.1007/s00394-024-03532-6.

## Introduction

Cardiovascular disease (CVD) remains globally the leading cause of death and the primary risk factor for the development of CVD is atherosclerosis and the subsequent pathogenic vascular calcification [1]. An important inhibitor of vascular calcification is matrix Gla protein (MGP), a vitamin K dependent protein (VKDP). A beneficial role of vitamin K on the progression of atherosclerosis has been suggested due to the presence of MGP in the vessel wall.

Vascular calcification is an important pathogenetic aspect of atherosclerosis; a complex process of calcification, fat depositing and inflammation causing a build-up of plaque lesions in the vessel wall. The pathogenesis is implicated by several different cardiovascular disease risk factors (CVDRFs) including hypertension, arterial stiffness, central obesity, diabetes, hyperlipidaemia, and impaired kidney function, causing damage to the endothelial layer, and depositing of calcium in the vessel wall. Especially in the coronary arteries, calcification is a strong predictor of future cardiovascular events [2]. An important inhibitor of vascular calcification is *carboxylated* MGP [3], predominantly synthesised in the medial layer of the vessel wall. MGP is carboxylated/activated by γ-glutamate carboxylase, for which vitamin K functions as an essential co-enzyme. Low vitamin K levels will lead to less activated MGP and cause an increase in the non-functional form, dephosphorylated and uncarboxylated MGP (dp-ucMGP) [4]. Intervention trials have found dp-ucMGP to decrease during vitamin K supplementation. In the present study, plasma dp-ucMGP is used as an inverse biomarker of functional vitamin K status [5].

Vitamin K is a fat-soluble vitamin, and includes two groups, K1, phylloquinone, and K2, menaquinones (MKs), the latter varying in length of the isoprenoid sidechains forming the vitamers MK4-MK13. K1 is mainly found in green vegetables whereas the K2s are found in animal based or fermented foods [6]. Most known and studied VKDPs are the hepatic proteins, responsible for activating coagulation factors but several extrahepatic VKDPs have essential functions. These include the anti-calcifying properties of MGP, predominantly preventing the medial calcification associated with loss of arterial compliance and conditions such as chronic kidney disease and diabetes as well as a strong prediction for future cardiovascular mortality [7, 8]. Another VKDP is the anti-inflammatory GAS6, inhibiting NF-κB transcription factors causing vascular inflammation in atherosclerotic plaques [9]. These functions support a biologically plausible protective effect of high vitamin K intake on the development of vascular calcification, inflammation, and atherosclerosis via multiple vitamin K dependent proteins [9]. Similarly, the opposite effect is observed with vitamin K antagonists (VKA), inhibiting the recycling of vitamin K. As the antagonists interfere with the γ-carboxylation of MGP, the regulation of soft tissue is impaired, subsequently promoting vascular calcification [10].

However, studies have found conflicting results regarding how vitamin K levels influence atherosclerosis, CVD, and CVDRFs. A meta-analysis of observational studies including 11 studies and 33,289 patients found that high dp-ucMGP was associated with 77% increased all-cause mortality (hazard ratio (HR) 1.77; 95% confidence interval (CI) 1.44–2.18), 84% increased CVD mortality (HR 1.84; 95% CI 1.44–2.18) and 41% increased risk of CVD (HR 1.41; 95% CI 0.94–2.12) [11]. When examining the association of dp-ucMGP and CVDRFs, a Danish population-based study of 491 subjects from 2020 found an association between higher dp-ucMGP and increased prevalence of central obesity (odds ratio (OR) 2.27; 95% CI 1.54–3.33) and arterial stiffness (OR 1.54; 95% CI 1.21–1.96), but no association with hypertension^13^. In chronic kidney disease patients both vitamin K deficiencies and significantly increased vascular calcification have been observed in the population [12]. A systematic review of randomised controlled trials (RCTs) investigating the effect of vitamin K supplements found inconsistent results regarding vitamin K’s role on arterial stiffness or vascular calcification, mainly detecting beneficial effects in predisposed groups [13]. Hence, we currently see *some* associations in observational studies, but further studies are needed to determine the association between vitamin K status and CVDRFs.

In this population-based cross-sectional study, we aimed to explore the association between vitamin K status measured using the biomarker dp-ucMGP and the CVDRFs implicated in the pathogenesis of atherosclerosis.

## Methods

### Data sources and population

**Fig. 1:**
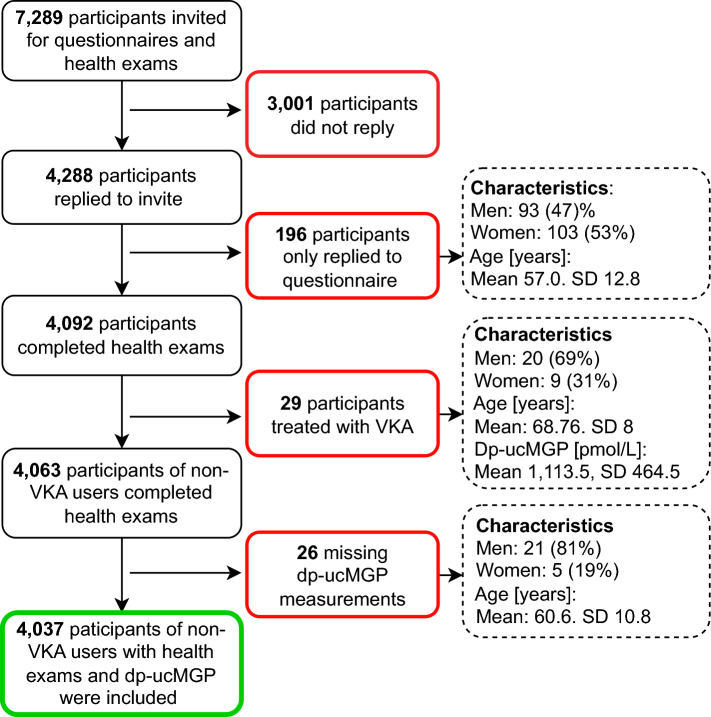
Flowchart of the inclusion and exclusion

This study is based on data from the 5-year follow-up of the Danish study of Functional Disorders (DanFunD), a population-based study comprising a randomly selected sample of adults residing in the Western part of Greater Copenhagen. Exclusion criteria included not being born in Denmark, non-Danish citizenship, or pregnancy [14]. A total of 7,493 individuals participated in the baseline study (29.5% of the invited). In 2018–2020 all eligible participants from baseline (N = 7,289) were invited and 4,092 (56.1%) participated in the follow-up examination carried out at the Center for Clinical Research and Prevention, Denmark. To obtain data on the use of VKA, all participants were linked to the Danish National Prescription Register via the unique Danish personal identification number [15]. The register contains information on all redeemed prescriptions from Danish pharmacies classified according to the Anatomical Therapeutic Chemical system (ATC-codes), including medication dose, quantity, and date of claim. In keeping with the known effect of VKA on the function of VKDPs, participants in active VKA treatment within 180 days prior to the health examination were excluded from the study (n = 29). Active VKA treatment was defined as redeemed prescriptions of VKA (ATC B01AA) of a minimum of 7 tablets per week. Participants with > 100 days between latest claim and study visit were not assessed as in active VKA treatment if they only had claimed one package containing 100 tablets [16]. Participants without measurements of dp-ucMGP (n = 26) were excluded from the analyses (Fig. 1).

### Exposure

Biochemical analysis of dp-ucMGP was performed according to the manufacturer’s instructions using the IDS-iSYS InaKtif MGP assay on the IDS-iSYS Multi-Discipline Automated System (Immunodiagnostic systems, plc, Tyne and Wear, UK) which is an in vitro diagnostic test intended for the quantitative determination of the inactive isoform of MGP, i.e., dp-ucMGP, in human plasma. Dp-ucMGP values below the lower limit of quantitation (<300 pmol/L) were set to 299 pmol/L (N = 135). Venous blood samples were drawn after > 6 h of fasting and collected using potassium EDTA tubes and separated by centrifugation at 2,000 g for 10 min at 4°C. Plasma was separated and stored at minus 20℃ within 90 min after blood sampling.

### Outcomes

Primary dichotomised outcomes included hypertension, high arterial stiffness, central obesity, diabetes, hyperlipidaemia, and impaired kidney function. Blood pressure was measured as systolic and diastolic blood pressure [mmHg] in two succeeding electronic measurements in sitting position after a minimum of five minutes of rest. The second measurement was included in the analysis. Hypertension was defined as systolic blood pressure > 140 mmHg or a diastolic blood pressure > 90 mmHg or self-reported use of antihypertensive medication. For the continuous outcomes, 15 mmHg and 10 mmHg were added to the systolic and diastolic measurement, respectively, if the participant reported taking antihypertensive medication or being diagnosed with hypertension [17]. Arterial stiffness was *estimated* using an index of aortic *Pulse Wave Velocity* (ePWV), and ePWV > 10 m/s was defined as high arterial stiffness based on the corrected systolic and diastolic measurement. Waist circumference was measured midway between the lower rib curvature and the iliac crest midaxillary and a circumference of ≥ 94 cm for men and ≥ 80 cm for women was defined as ‘central obesity’. Diabetes was defined as Haemoglobin A1C (HbA1c) > 48 mmol/mol or reporting “receiving treatment for diabetes”.

When the outcomes were used as continuous measurements, they were corrected for treatment: 7.5 mmol/mol were added to HbA1c measurement if the participant reported “receiving treatment for diabetes”[18]. Hyperlipidaemia was defined as Low Density Lipids (LDL) > 3 mmol/L, triglycerides > 2 mmol/L, total p-cholesterol > 5 mmol/L, or if they reported “receiving treatment for hyperlipidaemia”. For the continuous outcomes, 1.85 mmol/L, 1.63 mmol/L and 0.88 mmol/L were added to cholesterol, LDL, and triglyceride measurements, respectively, if the participant reported “receiving treatment for hyperlipidaemia” [19]. Kidney function was calculated using Chronic Kidney disease Epidemiology Collaboration creatinine equation and assessed as the estimated glomerular filtration rate (eGFR). Kidney disease was defined as eGFR < 60 mL/min. HbA1c, lipids and creatinine were analysed from freshly collected plasma and analysed with colorimetric slide methods using VITROS Chemistry Products. All biochemical analyses were performed at Department of Clinical Biochemistry, Rigshospitalet, Glostrup, Denmark.

### Covariates

Lifestyle factors were considered as covariates and included: diet, smoking status, leisure time physical activity level [20], and alcohol consumption. Information on fat percentage, Body Mass Index (BMI), and a full lipid status were included as descriptive variables. BMI was calculated from height and weight measurements. Fat percentage was measured via bioelectric body impedance using Tanita body analysis weight BC-420 MA. Information on alcohol consumption and smoking was collected using self-reported questionnaires and assessed as units per week for alcohol and as daily, occasional, previous, or never for smoking. The dietary habits were assessed using a self-reported characterisation of weekly food intake and scored using a healthy food index (HFI) to determine the diet quality [21]. Physical activity assessment was based on self-reported leisure time physical activity levels ranging from sedentary to moderate/high [20]. Information of previous AMI was assessed via self-reported questionnaires.

### Data analyses

The data was analysed using R Studio, version 4.3.2. The characteristics of the population were presented by quartiles of dp-ucMGP. Continuous variables were tested for normal distribution using Kolmogorov–Smirnov test and dp-ucMGP and blood pressures were log2-transformed to achieve approximate normal distributions.

The associations between dp-ucMGP and the primary outcomes, dichotomised CVDRFs, were estimated using multivariable logistic regression models with CVDRFs as the outcome variable and log2-transformed dp-ucMGP as the exposure variable. Results were expressed as odds ratios per doubling in dp-ucMGP levels. The associations were further tested using multivariable linear regression models with the continuous CVDRFs as the outcome variables and log2-transformed dp-ucMGP as exposure presented as change of CVDRFs per doubling in dp-ucMGP levels. Data was presented unadjusted and adjusted in three models: 1st adjusted for age and sex, 2nd further adjusted for waist circumference, smoking status, leisure time physical activity levels, alcohol consumption and diet, 3rd further adjusted for kidney function. Due to expected multicollinearity, fat percentage and BMI were not adjusted for waist circumference. Potential confounders were chosen based on their a priori presumed association with CVD and dp-ucMGP.

Sensitivity analyses were performed to assess whether 1) excluding outliers of dp-ucMGP (levels > 3 SD from the mean), 2) excluding participants who reported a history of AMI, 3) assessing dp-ucMGP as a dichotomic outcome (> 500 and < 500 pmol/L), 4) not correcting for treatment of hyperlipidaemia and diabetes affected the results. Subgroup analyses were performed for participants with hypertension, central obesity, low kidney function and aged > 65 years old to assess for any interactive effects analysed as the joint effect size. A p-value of less than 0.05 was the cut-off for statistical significance.

### Ethics

In the DanFunD study, written informed consent was obtained from each participant before participation, and the study was approved by the Ethical Committee of Copenhagen County (Ethics Committee: H-3–2012-0015 + H-17021141) and the Danish Data Protection Agency.

## Results

A total of 4,037 participants were included in the study. Characteristics of the population are presented according to dp-ucMGP quartiles: < 422, 423–504, 505–604 and > 604 pmol/L in Table 1. The mean dp-ucMGP was 528.1 pmol/L with a standard deviation (SD) of 162.3 in the total population and a range from 299 pmol/L to 2,330 pmol/L. The mean age for the population was 59.4 years, SD 11.6.

**Table 1:** Characteristics for the study population stratified by quartiles, Q1-Q4 of dp-ucMGP levels

	Q1_1_: < 422 pmol/L N = 1,028^1^	Q2_1_: 423-504 pmol/L N = 1,019^1^	Q3_1_: 505-604 pmol/L N = 1,020^1^	Q4_1_: > 604 pmol/L N = 970^1^
Dp-ucMGP [pmol/L]	363 ± 40	465 ± 23	552 ± 29	744 ± 162
Age [years]				
24–37	70 (6.8%)	62 (6.1%)	33 (3.2%)	24 (2.5%)
37–51	214 (21%)	178 (17%)	152 (15%)	89 (9.2%)
51–66	463 (45%)	455 (45%)	440 (43%)	399 (41%)
66–77	281 (27%)	324 (32%)	395 (39%)	458 (47%)
Sex				
Male (N = 1872)	450 (44%)	462 (45%)	503 (49%)	457 (47%)
Female (N = 2165)	578 (56%)	557 (55%)	517 (51%)	513 (53%)
Physiological markers				
Systolic Blood Pressure [mmHg]	127 ± 18	128 ± 18	130 ± 18	133 ± 18
Diastolic Blood Pressure [mmHg]	79 ± 11	78 ± 10	80 ± 11	80 ± 10
Estimated Pulse Wave Velocity [m/s]	9.14 ± 1.96	9.33 ± 1.98	9.69 ± 1.87	10.14 ± 1.78
Fat Percentage [%]	29 ± 8	29 ± 9	31 ± 9	33 ± 9
Body Mass Index [kg/m^2^]	25.2 ± 3.9	25.5 ± 4.0	26.8 ± 4.4	27.9 ± 5.1
Waist circumference [cm]	87 ± 12	88 ± 13	93 ± 13	96 ± 14
Anamnestic				
Alcohol consumption [N/week]	7 ± 7	8 ± 8	8 ± 9	8 ± 8
Healthy Food Index				
Unhealthy	86 (8.6%)	104 (11%)	133 (13%)	159 (17%)
Medium healthy	698 (70%)	688 (70%)	707 (72%)	643 (70%)
Healthy	211 (21%)	186 (19%)	147 (15%)	123 (13%)
Smoking status				
Daily	85 (8.3%)	74 (7.3%)	77 (7.6%)	62 (6.5%)
Occupational	37 (3.6%)	41 (4.0%)	29 (2.9%)	20 (2.1%)
Previous	376 (37%)	385 (38%)	415 (41%)	420 (44%)
Never	529 (52%)	514 (51%)	495 (49%)	459 (48%)
Daily use of tobacco [N/day]	12 ± 7	15 ± 11	14 ± 8	12 ± 8
Leisure time physical activity level				
Sedentary	72 (7%)	87 (9%)	110 (11%)	134 (14%)
Low	537 (52%)	561 (55%)	599 (59%)	584 (61%)
Moderate to high	418 (41%)	366 (36%)	307 (30%)	243 (25%)
Active antihypertensive treatment	208 (20%)	217 (21%)	264 (26%)	335 (35%)
Active antidiabetic treatment	26 (2.5%)	25 (2.5%)	38 (3.7%)	72 (7.5%)
Active cholesterol lowering treatment	160 (16%)	166 (16%)	207 (20%)	259 (27%)
History of acute myocardial infarction	14 (1.4%)	13 (1.3%)	33 (3.2%)	31 (3.2%)
Biomarkers				
Calcium [mmol/L]	2.34 ± 0.08	2.34 ± 0.08	2.34 ± 0.09	2.36 ± 0.09
Cholesterol, total [mmol/L]	5.20 ± 1.03	5.28 ± 1.01	5.30 ± 1.03	5.35 ± 1.13
HBA1c [mmol/mol]	37.1 ± 5.2	37.5 ± 4.5	38.0 ± 5.4	39.3 ± 7.3
High Density Lipids [mmol/L]	1.62 ± 0.48	1.60 ± 0.50	1.51 ± 0.46	1.50 ± 0.47
Low Density Lipids [mmol/L]	3.07 ± 0.92	3.14 ± 0.87	3.21 ± 0.95	3.22 ± 1.02
Triglycerides [mmol/L]	1.13 ± 0.66	1.18 ± 0.67	1.31 ± 0.78	1.47 ± 1.15
Very low-density lipids [mmol/L]	0.50 ± 0.26	0.52 ± 0.26	0.58 ± 0.30	0.63 ± 0.31
Kidney function [eGFR, mL/min/1.73m^2^]	84 ± 9	83 ± 10	82 ± 11	79 ± 13

*Dp-ucMGP* Dephosphorylated uncarboxylated Matrix Gla Protein 1: Mean and standard deviation or numbers and proportions in %

**Fig. 2:**
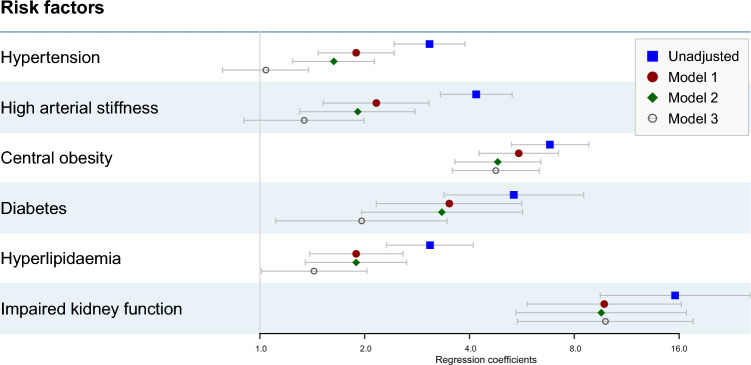
Odds-ratios and 95% confidence intervals from multivariable logistic regression models for the association between cardiovascular disease risk factors and vitamin K status (per doubling in dp-ucMGP) in all four models_1_

In the logistic regression analysis, a doubling of dp-ucMGP was associated with increased risk of central obesity (OR 4.76, 95% CI 3.57–6.34), diabetes (OR 1.96, 95% CI 1.11–3.45), hyperlipidaemia (OR 1.43, 95% CI 1.01–2.03), and kidney disease (OR 9.83, 95% CI 5.49–17.59) both in fully and partly adjusted models (Fig. 2, Supplementary Table 1). In partly adjusted models, hypertension (OR 1.04, 95% CI 0.78–1.38) and high arterial stiffness (OR 1.34, 95% CI 0.90–1.99) were positively associated with increasing levels of dp-ucMGP, however the associations attenuated when adjusted for further relevant covariates in model 3.

Similar associations were found between dp-ucMGP and the continuous CVDRFs, with a significant association between increasing dp-ucMGP levels and higher BMI, fat percentage, waist measurements, HbA1c-levels, total cholesterol-levels, LDL-levels, and triglycerides-levels across all models (Table 2). Furthermore, across all models, kidney function was found to decrease significantly per doubling in dp-ucMGP. Increasing dp-ucMGP was associated with increasing systolic blood pressure, diastolic blood pressure and ePWV, but the associations attenuated in the fully adjusted models. When assessing for possible interaction effects in the fully adjusted logistic regression models, only hypertension and age were found to have a statistically significant interactive effect. Hypertension increased the effect of dp-ucMGP on the risk of impaired kidney function (hypertension: OR 15.9, 95% CI 7.81–33.1 vs no hypertension: OR 3.61, 95% CI 1.33–9.55). Age was found to have a significantly higher effect of increasing dp-ucMGP levels on the risk of hyperlipidaemia for age below 65 years (OR 2.24, 95% CI 1.51–3.32) than age above 65 years (OR 0.61, 95% CI 0.33–1.14) (Supplementary Table 2).

**Table 2:** Estimated change and 95% confidence interval (CI) of cardiovascular disease risk factors per doubling in dp-ucMGP unadjusted and adjusted in three models. Blood pressure, blood sugar and lipids variables are corrected according to the estimated effect of treatment

	Unadjusted	Model 1	Model 2	Model 3
	Estimate [95% CI]	Estimate [95% CI]	Estimate [95% CI]	Estimate [95% CI]
Systolic blood pressure [mmHg]^1^	12.5 [10.2–14.9]	5.69 [3.49–7.89]	4.83 [2.49–7.17]	1.38 [−1.0–3.77]
Diastolic blood pressure [mmHg]^2^	4.04 [2.67–5.42]	2.08 [0.74–3.41]	1.57 [0.14–3.0]	−0.86 [−2.29–0.58]
Estimated Pulse Wave Velocity [m/s]^1,2^	1.68 [1.45–1.92]	0.40 [0.26–0.53]	0.34 [0.2–0.48]	0.06 [-0.08–0.21]
Body Mass Index [kg/m^2^]	3.89 [3.40–4.37]	3.59 [3.10–4.08]	3.12 [2.61–3.62]	3.03 [2.52–3.54]
Fat Percentage [%]	6.14 [5.18–7.11]	5.53 [4.80–6.26]	4.70 [3.94–5.45]	4.57 [3.81–5.33]
Waist circumference [cm]	13.5 [12.1–15.0]	11.3 [9.97–12.5]	9.74 [8.42–11.1]	9.65 [8.32–11.0]
HBA1c [mmol/mol]^3^	3.52 [2.79–4.26]	2.17 [1.44–2.89]	2.16 [1.41–2.90]	0.93 [0.17–1.70]
Cholesterol, total [mmol/L]^4^	0.48 [0.37–0.60]	0.24 [0.13–0.35]	0.25 [0.14–0.37]	0.17 [0.05–0.29]
Low density lipids [mmol/L]^5^	0.43 [0.33–0.53]	0.27 [0.18–0.37]	0.28 [0.18–0.38]	0.18 [0.08–0.29]
Triglycerides [mmol/L]^6^	0.61 [0.51–0.72]	0.48 [0.38–0.59]	0.47 [0.36–0.58]	0.19 [0.09–0.30]
Kidney function [eGFR, mL/min/1.72m^2^]	−7.32 [−8.49–(−6.14)]	−4.53 [-5.64–(−3.41)]	−4.45 [−5.64–(−3.27)]	−4.30 [−5.51–(−3.08)]

*Dp-ucMGP* Dephosphorylated uncarboxylated Matrix Gla Protein

Model 1: adjusted for sex and age groups

Model 2: Model 1 and in addition adjusted for smoking status, leisure time physical activity level, alcohol consumption and diet

Model 3: Model 2 and in addition adjusted for kidney function and waist circumference. Fat percentage, waist circumference and BMI are not adjusted for waist circumference

Variables corrected for treatment: 1: + 15 mmHg 2: + 10 mmHg 3: + 7.5 mmol/mol 4: + 1.85 mmol/L 5: + 1.63 mmol/L: 6: + 0.88 mmol/L

The results were essentially similar when excluding dp-ucMGP values of more than + 3SD from the mean (N = 60, Supplementary Table 3A and 3B) and results of the analyses dichotomising dp-ucMGP above and below 500 pmol/L were also reasonably consistent with non-dichotomised dp-ucMGP analyses (Supplementary Table 4A and 4B). Analyses performed when excluding participants who reported a history of AMI did not notably differ (Supplementary Table 5A and 5B), and nor did disregarding the effects of cholesterol lowering or antidiabetic treatment affect the estimated associations (Supplementary Table 6).

## Discussion

In this cross-sectional observational population-based study, we found associations between low vitamin K status assessed in this study as high dp-ucMGP levels and diabetes, hyperlipidaemia, and impaired kidney function. Furthermore, we confirmed the previous finding of a strong and independent association between higher dp-ucMGP levels and obesity [22]. A cross-sectional study in adults, investigating obesity and vitamin K biomarkers found significantly higher concentrations of vitamin K, in visceral fat tissue and lower circulating vitamin K status to be associated with increasing adiposity [23]. Consistent with this, an RCT in 214 postmenopausal women found that vitamin K supplementation for three years significantly decreased abdominal and visceral fat [24]. These findings suggest that vitamin K could be involved in central obesity. Since most of these results stem from observational studies, the causal direction of the associations cannot be fully determined. The results could be explained by an unhealthier diet in individuals with obesity and a decreased intake of green leafy vegetables, causing a lower intake of vitamin K. It should though also be considered that dp-ucMGP levels are likely also influenced by intakes of menaquinones (vitamin K2) from animal-based foods such as cheese and meats, which are considered less healthy foods in relation to cardiovascular risk. Currently, there is limited knowledge on the content of menaquinones in foods and thus intakes in the population [25]. In our study, adjusting for diet had no major effect on the association between dp-ucMGP and central obesity, supporting the hypothesis that the found association was not confounded nor mediated by dietary patterns. We did, however, find high dp-ucMGP levels to be tracking an unhealthy diet (Table 1).

As carboxylation of MGP is essential for MGP to inhibit arterial calcification, increased levels of dp-ucMGP may reflect decreased capacity to prevent the progression of arterial stiffening. This correlation was confirmed in a three-year RCT, discovering an improved arterial stiffness in the group of postmenopausal women receiving vitamin K supplementation [26]. In our observational study, we found an association between dp-ucMGP and arterial stiffness and hypertension, however the association attenuated and became statistically non-significant when adjusting for confounders such as age, kidney function, and waist circumference. When considering the pathophysiology of hypertension and arterial stiffness, the pathogenesis is mainly driven by increased vasoconstriction, disturbances in the intravascular fluids and increased sympathetic response. MGP is believed to mainly play a role in protection against calcification of tissues. Our finding of an apparent lack of association between dp-ucMGP and blood pressure might be explained by the expected effect of MGP, and hence dp-ucMGP, on calcification, a pathogenesis *following* hypertension rather than *causing* hypertension. As the arterial stiffness in our study was estimated using blood pressure measurements and age, it is not surprising that similar associations are observed for this variable. However, we did find the association between higher dp-ucMGP levels and impaired kidney function to be enhanced in participants with hypertension. In line with these findings, previous observational studies have found both subclinical vitamin K deficiency as well as an increased prevalence of medial arterial calcification in patients with chronic kidney disease [27]. As the vitamin K dependent MGP is mainly affecting the calcification in the medial layer of the artery wall, these findings could support the hypothesis that vitamin K could have anti-calcifying and protective properties important for the kidney function. An RCT in renal transplant patients treated with 360 µg K2 found a significant reduction in arterial stiffness over an 8-week period, though most clinical trials in patients and general populations have failed to show an effect of vitamin K supplementation on arterial stiffness [28, 29].

In the present study, we found a mean dp-ucMGP of 528.1 pmol/L with a range of 299 pmol/L to 2,330 pmol/L, which is in line with previous studies [22, 30]. We assessed the vitamin K status as dp-ucMGP, a generally accepted biomarker of functional vitamin K status, due to the known role of vitamin K for the carboxylation of MGP and hence lowering of the dp-ucMGP concentration. Several intervention studies have found vitamin K supplementation to lower the circulating dp-ucMGP concentration dose-dependently [31–34]. Other species of MGP include the functional forms, such as ucMGP or t-ucMGP but studies have not found these forms to be affected by vitamin K supplementation or use of VKA and are hence not considered valid markers for vitamin K status. However, the currently used marker, dp-ucMGP is affected by both the total MGP as well as local rates of synthesis and only represents a minor fraction of total MGP, which we are not able to assess [7]. Hence, we risk not assessing the genuine value of the functional VKDPs. To accommodate for the missing information on total MGP, we adjusted for age, based on the known association between increased total MGP and increasing age. Though as MGP is found to increase with age similarly as the health decreases and the risk of CVD increases with age, our found associations with dp-ucMGP could be due to the biomarker simply being a trait of poor health. This is also observed in the distribution of dp-ucMGP in our descriptive statistics, with increasing dp-ucMGP levels following a less active lifestyle. However, similarly as seen when adjusting for diet, no major effects were observed when we adjusted our analyses for leisure time physical activity levels.

Several limitations and strengths in this observational study should be addressed. As our study is based on a cross-sectional design, it is unable to depict causal inference and prone to reverse causation. Hence, we cannot reject whether our found associations are in fact caused by an increased local synthesis of dp-ucMGP because of a pathological process in the cardiovascular system. However, either causation support the hypothesis of an increased need of vitamin K to prevent progression in CVDRFs. To account for the limitation that factors affecting the dp-ucMGP levels are largely unknown, we conducted four different adjustment models, hereby risking both overadjustment and residual confounding but were also able to assess the associations on various levels.

As a strength, we were able to link the population to public health care registers and account for active treatment with VKA. We further considered the self-reported use of both antihypertensive, antidiabetic and lipid lowering treatment and corrected our analyses according to treatment effects from large observational studies thus assessing the underlying and untreated values. However, as the method of adjusting for other treatments than antihypertensive treatment is not commonly used despite substantial risk of skewness in data if avoided, no well-established values were available for corrections. Furthermore, by using self-reported information, we increased the risk of recall-bias. On the other hand, several Danish studies have found self-reported data to be accurate and representative in comparison to register data when assessing diagnoses and use of medication [35]. Lastly, we assessed the levels using the circulating biomarker rather than solely using estimates based on self-reported intake. Thus, we avoided recall bias as well as the inherent limitations currently present in estimating dietary intakes of vitamin K from dietary questionnaires, e.g., due to lack of data on MKs in foods [6] However, by not having estimates on vitamin K intake available, we were unable to validate our biomarker as a measure of vitamin K status.

In conclusion, we found independent associations between dp-ucMGP levels and central obesity, diabetes, hyperlipidaemia, and impaired kidney function. We found no independent associations between dp-ucMGP levels and hypertension or estimated pulse wave velocity (arterial stiffness). We did find a stronger association between dp-ucMGP levels and impaired kidney function for hypertensive participants than for normotensive participants. These results suggest that, if vitamin K has an influence on cardiovascular disease, the complex mechanisms may involve obesity, diabetes, and kidney function. Larger randomised controlled trials are needed to assess whether vitamin K is causally related to these cardiovascular disease risk factors both in predisposed groups and in the general population.

## Supplementary Information

Below is the link to the electronic supplementary material.

Supplementary file1 (PDF 325 KB)

## Data Availability

The dataset supporting the conclusions of this article is based on data from Danish national health registers and restrictions apply to the availability of these data, which were accessed through Statistics Denmark’s server under license for the current study. According to Danish law, this information cannot be publicly available. A request for access to the data needs approval from appropriate Danish authorities and are subject to Danish regulations on personal data protection. The main dataset is hosted at Center for Clinical Research and Prevention [ckff@regionh.dk].
