# Supplementary material for: Investigating the associations between uncarboxylated matrix gla protein as a proxy for vitamin K status and cardiovascular disease risk factors in a general adult population
Source: Eur J Nutr. 2024 Nov 21;64(1):17. doi: 10.1007/s00394-024-03532-6 (PMC11579057; doi:10.1007/s00394-024-03532-6)
Supplement: Supplementary file 1 — Supplementary file1 (PDF 325 KB) [file 394_2024_3532_MOESM1_ESM.pdf]

Supplementary

Article title: The association between vitamin K status and cardiovascular disease risk factors in a general adult population

Journal name: European Journal of Nutrition

Author names: Julie Aaberg Lauridsen, Katja Biering Leth-Møller, Line Tang Møllehave, Line Lund Kårhus, Thomas Meinertz Dantoft, Klaus Fuglsang Kofoed, Allan Linneberg

Corresponding author: Julie Aaberg Lauridsen, Center for Clinical Research and Prevention, Copenhagen University Hospital, Bispebjerg and Frederiksberg hospital, Denmark

E-mail: julie.aaberg.lauridsen@regionh.dk

Supplementary table 1 Odds-ratios (ORs) and 95% confidence intervals (CIs) from multivariable logistic regression models for the association between cardiovascular disease risk factors and vitamin K status per doubling in dp-ucMGP unadjusted and adjusted in three models

	**%**	**Unadjusted**	**Model 1**	**Model 2**	**Model 3**
	n/Total N	OR [95% CI]	OR [95% CI]	OR [95% CI]	OR [95% CI]
**Hypertension**	1,689/4,027	3.07 [2.43–3.88]	1.89 [1.47–2.43]	1.63 [1.24–2.13]	1.04 [0.78–1.38]
**High arterial stiffness**	1,766/4,036	4.18 [3.30–5.30]	2.16 [1.52–3.06]	1.91 [1.30–2.79]	1.34 [0.90–1.99]
**Central obesity**	2,464/4,033	6.81 [5.28–8.80]	5.54 [4.26–7.20]	4.82 [3.62–6.41]	4.76 [3.57–6.34]
**Diabetes**	212/4,015	5.36 [3.38–8.51]	3.50 [2.16–5.65]	3.33 [1.96–5.68]	1.96 [1.11–3.45]
**Hyperlipidaemia**	3,180/4,031	3.08 [2.31–4.10]	1.89 [1.39–2.58]	1.89 [1.35–2.64]	1.43 [1.01–2.03]
**Impaired kidney function**	181/4,037	15.58 [9.50–25.57]	9.75 [5.86–16.22]	9.56 [5.44–16.8]	9.83 [5.49–17.59]

Dp-ucMGP: Dephosphorylated uncarboxylated Matrix Gla Protein
Model 1: adjusted for sex and age as a categorial variable
Model 2: further adjusted for smoking status, leisure time physical activity level, alcohol consumption and diet
Model 3: further adjusted for kidney function and waist circumference. Central obesity and impaired kidney function were not adjusted for the defining variables

Subgroup analyses

Supplementary table 2 Interaction models, testing for the effect of central obesity, hypertension, impaired kidney function and age above 65 years. The effects are presented with estimated Odds ratio (OR), 95% confidence intervals (CI) and significance levels

	**Central obesity**			**Hypertension**			**Kidney disease**			**> 65 years old**		
	OR [95% CI]			OR [95% CI]			OR [95% CI]			OR [95% CI]		
	Yes	No	P-value	Yes	No	P-value	Yes	No	P-value	Yes	No	P-value
**Hypertension₁**	1.22 [0.87–1.70]	1.3 [0.79–2.13]	0.82	-	-		1.82 [0.63–5.61]	1.01 [0.75–1.36]	0.24	0.88 [0.58–1.34]	1.34 [0.93–1.93]	0.13
**High arterial stiffness₁**	1.45 [0.91–2.34]	1.62 [0.81–3.25]	0.80	-	-		4.41 [0.83–25.7]	1.28 [0.85–1.93]	0.16	1.34 [0.64–2.83]	1.56 [1.00–2.43]	0.73
**Central obesity₁**	-	-		3.57 [2.30–5.59]	5.63 [3.87–8.25]	0.12	6.50 [2.02–23.4]	4.80 [3.58–6.45]	0.63	3.68 [2.32–5.82]	6.32 [4.43–9.03]	0.07
**Diabetes₁**	2.55 [1.43–4.56]	2.79 [0.50–14.2]	0.92	1.72 [0.93–3.22]	2.57 [0.74–8.42]	0.46	9.49 [1.87–58.1]	1.35 [0.73–2.49]	0.04	1.65 [0.84–3.25]	2.88 [1.12–7.43]	0.34
**Hyperlipidaemia₁**	1.78 [1.12–2.85]	1.22 [0.75–2.00]	0.28	1.56 [0.88–2.80]	1.32 [0.87–2.02]	0.60	1.81 [0.31–11.7]	1.44 [1.02–2.05]	0.75	0.61 [0.33–1.14]	2.24 [1.51–3.32]	<0.05
**Impaired kidney function₁**	13.5 [6.96–26.7]	3.57 [1.10–11.2]	0.05	15.9 [7.81–33.1]	3.61 [1.33–9.55]	<0.05	-	-		11.47 [5.93–22.18]	6.62 [1.98–22.21]	0.43

1: Model adjusted for sex, age groups, smoking status, leisure time physical activity level, alcohol consumption, diet, kidney function and waist circumference. Models are not adjusted for interacting or defining variables.

Sensitivity analyses

Supplementary table 3A Odds-ratios (OR) and 95% confidence intervals (CI) from multivariable logistic regression models for the association between cardiovascular disease risk factors and vitamin K status per doubling in dp-ucMGP‡ unadjusted and adjusted in three models. Sensitivity analysis 1A: dp-ucMGP values +3 standard deviations from the mean are excluded.

	**%**	**Unadjusted**	**Model 1**	**Model 2**	**Model 3**
	n/Total N	OR [95% CI]	OR [95% CI]	OR [95% CI]	OR [95% CI]
**Hypertension**	1,653/ 3,967	3.24 [2.52–4.17]	2.01 [1.53–2.63]	1.75 [1.31–2.33]	1.08 [0.79–1.46]
**High arterial stiffness**	1,718/ 3,976	4.02 [3.12–5.17]	2.02 [1.38–2.94]	1.79 [1.20–2.69]	1.20 [0.79–1.83]
**Central obesity**	2,414/ 3,973	7.25 [5.55–9.47]	5.87 [4.46–7.72]	5.29 [3.93–7.11]	5.25 [3.90–7.06]
**Diabetes**	202/ 3,955	5.61 [3.22–9.75]	3.37 [1.92–5.93]	3.06 [1.64–5.69]	1.64 [0.86–3.13]
**Hyperlipidaemia**	3,129/ 3,971	3.29 [2.43–4.46]	2.01 [1.45–2.78]	2.06 [1.46–2.92]	1.53 [1.07–2.20]
**Impaired kidney function**	166/ 3,977	20.09 [10.70–37.72]	11.49 [6.05–21.82]	10.20 [5.09–20.41]	10.57 [5.15–21.70]

Dp-ucMGP: Dephosphorylated uncarboxylated Matrix Gla Protein.
Model 1: adjusted for sex and age as a categorial variable
Model 2: further adjusted for smoking status, leisure time physical activity level, alcohol consumption and diet
Model 3: further adjusted for kidney function and waist circumference. Central obesity and kidney disease were not adjusted for the defining variables.

Supplementary table 3B Estimated change and 95% confidence interval of cardiovascular disease risk factors per doubling in dp-ucMGP‡ unadjusted and adjusted in three models. Blood pressure, blood sugar and lipids variables are corrected according to the estimated effect of treatment. Sensitivity analysis 1B: dp-ucMGP values +3 standard deviations from the mean are excluded

	**Unadjusted**	**Model 1**	**Model 2**	**Model 3**
	Estimate [95% CI]	Estimate [95% CI]	Estimate [95% CI]	Estimate [95% CI]
**Systolic blood pressure [mmHg]₁**	12.9 [10.2–15.5]	5.95 [3.57–8.33]	5.16 [2.65–7.67]	1.46 [-1.09–4.01]
**Diastolic blood pressure [mmHg]₂**	3.97 [2.48–5.45]	1.88 [0.43–3.33]	1.52 [-0.01–3.06]	-1.21 [-2.75–[-0.33]]
**Estimated Pulse Wave Velocity [m/s]₁,₂**	1.70 [1.44 –1.95]	0.41 [0.26–0.55]	0.35 [0.20–0.51]	0.063 [-0.09–0.22]
**Body Mass Index [kg/m²]**	4.17 [3.64–4.69]	3.86 [3.33–4.39]	3.40 [2.86–3.94]	3.33 [2.78–3.88]
**Fat Percentage [%]**	6.54 [5.50–7.59]	5.87 [5.08–6.66]	5.14 [4.32–5.95]	5.03 [4.21–5.84]
**Waist [cm]**	14.1 [12.5–15.7]	11.8 [10.4–13.1]	10.4 [8.99–11.8]	10.4 [8.93–11.8]
**HBA1c [mmol/mol]₃**	3.48 [2.69–4.28]	2.10 [1.32–2.87]	2.02 [1.23–2.82	0.73 [-0.07–1.54]
**Cholesterol, total [mmol/L]₄**	0.538 [0.41–0.66]	0.28 [0.16–0.40]	0.31 [0.19–0.44]	0.22 [0.09–0.34]
**Low Density Lipids [mmol/L]₅**	0.49 [0.39–0.60]	0.33 [0.22–0.43]	0.35 [0.24–0.46]	0.24 [0.13–0.36]
**Triglycerides [mmol/L]₆**	0.54 [0.44–0.64]	0.41 [0.31–0.51]	0.39 [0.29–0.49]	0.11 [0.01–0.21]
**Kidney function [eGFR, mL/min/1.72m²]**	-7.25 [-8.51–(-5.99)]	-4.49 [-5.69–(-3.30)]	-4.24 [-5.49–(-2.99)]	-4.15 [-5.43–(-2.86)]

Dp-ucMGP: Dephosphorylated uncarboxylated Matrix Gla Protein.
Model 1: adjusted for sex and age groups
Model 2: Model 1 and in addition adjusted for smoking status, leisure time physical activity level, alcohol consumption and diet.
Model 3: Model 2 and in addition adjusted for kidney function and waist circumference. Fat percentage, waist circumference and BMI are not adjusted for waist circumference.
Variables corrected for treatment: 1: +15mmHg 2: +10mmHg 3: +7.5mmol/mol 4: +1.85mmol/L 5: +1.63mmol/L: 6: +0.88mmol/L

Supplementary table 4A: Odds-ratios and 95% confidence intervals from multivariable logistic regression models for the association between cardiovascular disease risk factors and vitamin K status assessed as dp-ucMGP‡ unadjusted and adjusted in three models. Sensitivity analysis 2A: Dp-ucMGP values are dichotomised: <500 and >500pmol/L.

	**%**	**Unadjusted**	**Model 1**	**Model 2**	**Model 3**
	n/Total N	OR [95% CI]	OR [95% CI]	OR [95% CI]	OR [95% CI]
**Hypertension**	1,689/4,027	1.68 [1.48–1.90]	1.33 [1.16–1.53]	1.30 [1.12–1.51]	1.05 [0.90–1.23]
**High arterial stiffness**	1,766/4,036	1.79 [1.58–2.03]	1.24 [1.01–1.53]	1.25 [1.02–1.53]	1.03 [0.84–1.27]
**Central obesity**	2,464/4,033	2.49 [2.19–2.84]	2.25 [1.97–2.57]	2.14 [1.86–2.47]	2.13 [1.84–2.46]
**Diabetes**	212/4,015	2.26 [1.68–3.05]	1.78 [1.31–2.41]	1.63 [1.17–2.28]	1.26 [0.89–1.78]
**Hyperlipidaemia**	3,180/4,031	1.67 [1.43–1.94]	1.31 [1.11–1.54]	1.33 [1.12–1.59]	1.16 [0.97–1.39]
**Impaired kidney function**	181/4,037	3.22 [2.27–4.55]	2.47 [1.73–3.52]	2.44 [1.66–3.58]	2.37 [1.60–3.50]

Dp-ucMGP: Dephosphorylated uncarboxylated Matrix Gla Protein
Model 1: adjusted for sex and age as a categorial variable
Model 2: further adjusted for smoking status, leisure time physical activity level, alcohol consumption and diet
Model 3: further adjusted for kidney function and waist circumference. Central obesity and kidney disease were not adjusted for the defining variables

Supplementary table 4B: Estimated change and 95% confidence interval of cardiovascular disease risk factors per increase in dp-ucMGP category‡ unadjusted and adjusted in three models. Blood pressure, blood sugar and lipids variables are corrected according to the estimated effect of treatment. Sensitivity analysis 2B: Dp-ucMGP values are dichotomised: <500 and >500pmol/L.

	**Unadjusted**	**Model 1**	**Model 2**	**Model 3**
	Estimate [95% CI]	Estimate [95% CI]	Estimate [95% CI]	Estimate [95% CI]
**Systolic blood pressure [mmHg]$_1$**	5.61 [4.28–6.94]	2.58 [1.36–3.79]	2.36 [1.08–3.64]	0.701 [-0.58–1.99]
**Diastolic blood pressure [mmHg]$_2$**	2.11 [1.34–2.87	1.17 [0.43–1.92]	1.09 [0.31–1.88]	-0.12 [-0.90–0.66]
**Estimated Pulse Wave Velocity [m/s]$_{1,2}$**	0.74 [0.61–0.87]	0.18 [0.10–0.25]	0.16 [0.08–0.24]	0.03 [-0.05–0.11]
**Body Mass Index [kg/m²]**	1.88 [1.61–2.15]	1.72 [1.44–1.99]	1.51 [1.23–1.79]	1.47 [1.20–1.75]
**Fat Percentage [%]**	2.95 [2.40–3.49]	2.70 [2.30–3.11]	2.35 [1.93–2.77]	2.31 [1.89–2.72]
**Waist [cm]**	6.62 [5.81–7.44]	5.47 [4.75–6.19]	4.79 [4.06–5.51]	4.74 [4.01–5.47]
**HBA1c [mmol/mol]$_3$**	1.43 [1.02–1.84]	0.82 [0.415–1.22]	0.69 [0.28–1.09]	0.09 [-0.32–0.50]
**Cholesterol, total [mmol/L]$_4$**	0.22 [0.16–0.28]	0.11 [0.05–0.17]	0.13 [0.06–0.19]	0.08 [0.02–0.15]
**Low Density Lipids [mmol/L]$_5$**	0.22 [0.16–0.27]	0.14 [0.09–0.20]	0.16 [0.10–0.21]	0.11 [0.05–0.16]
**Triglycerides [mmol/L]$_6$**	0.28 [0.22–0.34]	0.22 [0.16–0.27]	0.20 [0.14–0.26]	0.07 [0.01–0.12]
**Kidney function [eGFR, mL/min/1.72m²]**	-2.90 [-3.56–(-2.24)]	-1.67 [-2.29–(-1.05)]	-1.53 [-2.17–(-0.90)]	-1.41 [-2.07–(-0.75)]

Dp-ucMGP: Dephosphorylated uncarboxylated Matrix Gla Protein
Model 1: adjusted for sex and age groups
Model 2: Model 1 and in addition adjusted for smoking status, leisure time physical activity level, alcohol consumption and diet.
Model 3: Model 2 and in addition adjusted for kidney function and waist circumference. Fat percentage, waist circumference and BMI are not adjusted for waist circumference.
Variables corrected for treatment: 1: +15mmHg 2: +10mmHg 3: +7.5mmol/mol 4: +1.85mmol/L 5: +1.63mmol/L: 6: +0.88mmol/L

Supplementary table 5A Odds-ratios and 95% confidence intervals from multivariable logistic regression models for the association between cardiovascular disease risk factors and vitamin K status assessed as dp-ucMGP‡ unadjusted and adjusted in three models. Sensitivity analysis 4A: Participants with a self-reported history of AMI are excluded

	**%**	**Unadjusted**	**Model 1**	**Model 2**	**Model 3**
	n/Total N	OR [95% CI]	OR [95% CI]	OR [95% CI]	OR [95% CI]
**Hypertension**	1,619/3,936	3.01 [2.38–3.80]	1.84 [1.43–2.37]	1.62 [1.23–2.12]	1.02 [0.77–1.36]
**High arterial stiffness**	1,716/3,945	4.22 [3.32–5.36]	2.14 [1.51–3.05]	1.95 [1.32–2.86]	1.34 [0.90–2.01]
**Central obesity**	2,388/3,942	6.70 [5.18–8.67]	5.41 [4.15–7.04]	4.77 [3.58–6.35]	4.73 [3.55–6.30]
**Diabetes**	197/3,924	5.64 [3.51–9.06]	3.69 [2.25–6.04]	3.44 [2.00–5.92]	1.95 [1.10–3.48]
**Hyperlipidaemia**	3,093/3,940	3.09 [2.32–4.13]	1.88 [1.38–2.56]	1.89 [1.35–2.63]	1.42 [1.00–2.00]
**Impaired kidney function**	171/3,946	15.37 [9.28–25.45]	9.65 [5.74–16.23]	9.73 [5.49–17.25]	9.77 [5.41–17.65]

Dp-ucMGP: Dephosphorylated uncarboxylated Matrix Gla Protein
Model 1: adjusted for sex and age as a categorial variable
Model 2: further adjusted for smoking status, leisure time physical activity level, alcohol consumption and diet
Model 3: further adjusted for kidney function and waist circumference. Central obesity and kidney disease were not adjusted for the defining variables

Supplementary table 5B Estimated change and 95% confidence interval of cardiovascular disease risk factors per doubling in dp-ucMGP‡ unadjusted and adjusted in three models. Blood pressure, blood sugar and lipids variables are corrected according to the estimated effect of treatment. Sensitivity analysis 4B: Participants with a self-reported history of AMI are excluded

	**Unadjusted**	**Model 1**	**Model 2**	**Model 3**
	Estimate [95% CI]	Estimate [95% CI]	Estimate [95% CI]	Estimate [95% CI]
**Systolic blood pressure [mmHg]$_1$**	12.6 [10.2–15.1]	5.77 [3.54–8.00]	4.98 [2.61–7.35]	1.44 [-0.98−3.85]
**Diastolic blood pressure [mmHg]$_2$**	4.02 [2.63–5.41]	1.95 [0.60–3.31]	1.59 [0.15–3.04]	-1.01 [-2.46−0.45]
**Estimated Pulse Wave Velocity [m/s]$_{1,2}$**	1.68 [1.45–1.92]	0.41 [0.27–0.55]	0.36 [0.21−0.50]	0.08 [-0.07−0.23]
**Body Mass Index [kg/m²]**	3.88 [3.39–4.36]	3.58 [3.09–4.08]	3.16 [2.65−3.67]	3.09 [2.57−3.60]
**Fat Percentage [%]**	6.11 [5.13–7.09]	5.47 [4.74–6.21]	4.72 [3.96−5.49]	4.63 [3.85−5.40]
**Waist [cm]**	13.5 [12.1–15.0]	11.2 [9.95–12.5]	9.91 [8.58–11.2]	9.86 [8.52−11.2]
**HBA1c [mmol/mol]$_3$**	3.42 [2.69–4.15]	2.09 [1.37–2.81]	2.05 [1.31−2.78]	0.85 [0.10−1.60]
**Cholesterol, total [mmol/L]$_4$**	0.50 [0.38–0.61]	0.24 [0.13–0.35]	0.27 [0.15–0.38]	0.17 [0.05−0.29]
**Low Density Lipids [mmol/L]$_5$**	0.45 [0.35–0.55]	0.27 [0.18–0.38]	0.30 [0.19–0.40]	0.18 [0.08−0.29]
**Triglycerides [mmol/L]$_6$**	0.61 [0.5–0.71]	0.479 [0.38–0.58]	0.47 [0.36–0.58]	0.20 [0.09−0.31]
**Kidney function [eGFR, mL/min/1.72m²]**	-7.23 [-8.41–(-6.05)]	-4.50 [-5.63–(-3.38)]	-4.50 [-5.69–(-3.32)]	-4.42 [-5.64− (-3.20)]

Dp-ucMGP: Dephosphorylated uncarboxylated Matrix Gla Protein
Model 1: adjusted for sex and age groups
Model 2: Model 1 and in addition adjusted for smoking status, leisure time physical activity level, alcohol consumption and diet.
Model 3: Model 2 and in addition adjusted for kidney function and waist circumference. Fat percentage, waist circumference and BMI are not adjusted for waist circumference.
Variables corrected for treatment: 1: +15mmHg 2: +10mmHg 3: +7.5mmol/mol 4: +1.85mmol/L 5: +1.63mmol/L: 6: +0.88mmol/L

Supplementary table 6 Estimated change and 95% confidence interval (CI) of cardiovascular disease risk factors per doubling in dp-ucMGP‡ unadjusted and adjusted in three models. Sensitivity analysis 3: Analyses are not corrected for use of cholesterol lowering medication and antidiabetic medication

	**Unadjusted**	**Model 1**	**Model 2**	**Model 3**
	Estimate [95% CI]	Estimate [95% CI]	Estimate [95% CI]	Estimate [95% CI]
**Systolic blood pressure [mmHg]**	12.50 [10.2–14.9]	5.69 [3.49–7.89]	4.83 [2.49–7.17]	1.38 [-1,0–3.77]
**Diastolic blood pressure [mmHg]**	4.04 [2.67–5.42]	2.08 [0.74–3.41]	1.57 [0.14–3.0]	-0.86 [-2.29–0.58]
**Estimated Pulse Wave Velocity [m/s]**	1.68 [1.45–1.92]	0.40 [0.26–0.53]	0.34 [0.2–0.48]	0.06 [-0.08–0.21]
**Body Mass Index [kg/m²]**	3.89 [3.40–4.37]	3.59 [3.10–4.08]	3.12 [2.61–3.62]	3.03 [2.52–3.54]
**Fat Percentage [%]**	6.14 [5.18–7.11]	5.53 [4.80–6.26]	4.70 [3.94–5.45]	4.57 [3.81–5.33]
**Waist [cm]**	13.5 [12.1–15.0]	11.3 [9.97–12.5]	9.74 [8.42–11.1]	9.65 [8.32–11.0]
**HBA1c [mmol/mol]**	3.01 [2.38–3.64]	1.79 [1.17–2.41]	1.80 [1.16–2.45]	0.74 [0.08–1.39]
**P-Cholesterol [mmol/L]**	0.18 [0.06–0.30]	0.10 [-0.02–0.21]	0.13 [0.01–0.25]	0.14 [0.02–0.27]
**Low Density Lipids [mmol/L]**	0.17 [0.07–0.28]	0.16 [0.05–0.27]	0.19 [0.07–0.30]	0.17 [0.05–0.29]
**Triglycerides [mmol/L]**	0.48 [0.39–0.57]	0.43 [0.33–0.52]	0.41 [0.31–0.51]	0.18 [0.08–0.28]
**Kidney function [eGFR, mL/min]**	-7.32 [-8.49–(-6.14)]	-4.53 [-5.64–(-3.41)]	-4.45 [-5.64–(-3.27)]	-4.30 [-5.51–(-3.08)]

Dp-ucMGP: Dephosphorylated uncarboxylated Matrix Gla Protein
Model 1: adjusted for sex and age groups
Model 2: Model 1 and in addition adjusted for smoking status, leisure time physical activity level, alcohol consumption and diet.
Model 3: Model 2 and in addition adjusted for kidney function and waist circumference. Fat percentage, waist circumference and BMI are not adjusted for waist circumference.
